# An Association of Quality of Life and Ageing Perceptions among Community Dwelling Older Adults in Uganda

**DOI:** 10.24966/ggm-8662/100142

**Published:** 2022-08-12

**Authors:** JL Gumikiriza-Onoria, R Odokonyero, B Giordani, D Akena, E Mwesiga, B Ssuna, SC Ray, RC Bollinger, NK Sewankambo, N Nakasujja

**Affiliations:** 1 Department of Psychiatry, School of Medicine, Makerere University College of Health Sciences, Kampala, Uganda; 2 University of Michigan, Ann Arbor, USA; 3 Department of Epidemiology and Biostatistics, Makerere College of Health Sciences, Kampala, Uganda; 4 Johns Hopkins University School of Medicine, Baltimore, USA; 5 Department of Medicine, School of Medicine, Makerere University College of Health Sciences, Kampala, Uganda

**Keywords:** Community dwelling older adults, Ageing perceptions, Quality of life

## Abstract

**Background:**

Uganda’s population, though, largely characterized by young people, has seen the number of people aged 60 and over grow from 686,000 twenty years ago, to 1,433,596 in 2014. Effective caring for the well-being of this population requires strategic and deliberate planning that involves Quality Of Life (QoL) assessments. QoL assessments among the elderly are important in evaluating the efficacy of strategies, such as health interventions, welfare programs, health care and well-being of the elderly. However, elderly in Uganda face several challenges, ranging from loneliness, poor housing, lack of social and financial support and poor health. These may negatively affect older persons’ quality of life and consequently their perceptions and attitudes towards aging.

**Methods:**

The study was carried out in 2019 in the communities of Nansana and Busukuma town councils in Wakiso district, Uganda. The participants were 380 people 60 years and older. To establish the association between perceptions of ageing and QoL, this study utilized a locally adapted version of the Older Person’s Quality of Life Questionnaire (OPQOL) and the Brief Ageing Perceptions Questionnaire (B-APQ). The OPQOL assesses three domains of QoL: Health QoL (HQoL); Social economic QoL (SQoL); and Psychosocial QoL (PQoL). The B-APQ assesses perceptions about physical age, participation in social activities, and perceptions about ability to regulate emotions as one ages. Pearson’s Chi-square tests were used to characterize the relationship between the perceptions and quality of life.

**Results:**

The majority of the respondents, 61% (95%CI 56.7–64.8), had negative perceptions towards ageing. Eighty six percent had poor HQoL, 90% poor SQoL and 83% poor PQoL. There was a significant association between good HQoL and positive perception about participation in social activities (X2 = 7.3670, P = 0.007) as well as with positive perception on regulation of emotions (X2 = 18.1803, P<0.001). There was a significant association between good SQoL and positive perception about participation in social activities (X2 = 5.3472, P = 0.021), as well with positive perception on regulation of emotions (X2 = 10.5128, P<0.001). A significant association between good PQoL and positive perception on regulation of emotions (X2 = 9.2414, P= 0.002).

**Conclusion:**

Positive perceptions of ageing are associated with good QoL. Directly addressing perceptions of ageing could be a low cost and effective strategy to improve the QoL of older persons in SSA.

## Introduction

Worldwide, there has been a noticeable increase in the population of the elderly [1]. Along with this demographic changes are renewed calls for global concern [2]. The United Nations defines the elderly as those aged 60 years and older [3]. The World Health Organization [4] has referred to this as a demographic revolution and together with the United Nations, predict that by 2025 the world population of the elderly will have reached 1.2 billion people, and doubled by 2050 [4,5]. This observed increase has mostly been attributed to improved health care systems, low fertility rates, advances in medicine and healthcare, and public health policies, among other reasons [6,7].

The WHO recommends that for aging to be a positive experience, there is need for good health, participation, and security [4]. Consequently, quite a bit of focus is recently being given to research that focuses on the well-being of the elderly population [8]. The well-being of the elderly population is understudied in developing countries like Uganda. Therefore, studies about quality of life among the elderly are essential. Uganda’s population, though, largely characterized by young people, has seen the number of people aged 60 and over grow from 686,000 twenty years ago, to 1,433,596 in 2014. Caring for the well-being of this population requires strategic and deliberate planning. Quality of life assessments among the elderly are important in evaluating the efficacy of some of these strategies like health interventions, welfare programs, health care and well-being of the elderly. However, older persons in Uganda face several challenges, ranging from loneliness, poor housing, lack of social and financial support and poor health [9–11]. These may negatively affect their quality of life and consequently their perceptions and attitudes towards aging.

The Social Assistance Grant for Empowerment (SAGE) for the elderly program has been a commendable effort by the Government of Uganda to respond to the socio-economic needs of the country’s elderly population [12]. However, any such programs and policies still fall short of improving quality of life among the elderly in Uganda. Moreover, Ugandan research studies on the elderly have mostly addressed issues such as; nutrition, vulnerability, functional ability and poverty [9,10,13,14]. Though these studies generated important information on the plight of the elderly, they were rather silent on the quality of life of the elderly in Uganda. Maniragaba et al., contributed to this important conversation regarding the quality of life of the elderly in Uganda, and reported that being HIV positive, the region of residence and household dynamics were predictors of poor quality of life [15]. Studies such as these however looked outside the individual for predictors of quality of life yet it is clear that how one views things in life are very important predictors of how they will behave, think and feel [16].

Though a few studies from the Western world have looked at perceptions towards aging and its influence on one’s quality of life [17], there are scarcely any such studies from Sub Saharan Africa and in particular in Uganda. To fill this knowledge gap, this study set out to determine the relationship between perceptions on aging and quality of life among the elderly in Central Uganda.

## Methods

### Study design and setting

This cross-sectional study was conducted between September 2017 to October 2018. The study was conducted in the communities of two sub-counties of Wakiso district. Wakiso is a district in Central Uganda that encircles Kampala, the capital city of Uganda. Wakiso district is currently one of three districts popularly referred to as the Greater Kampala Metropolitan District (KMD). This is due to their proximity to the Central Business District of Kampala Capital City. Wakiso is home to nearly 2 million people as of 2018, who live in a mixture of urban, suburban and rural settings. Approximately 92% of the district’s population lives in the rural areas of the district. Regard-less of the setting in which people live, the levels of poverty are significant throughout the district. The district has a population of more than 400,000 older people aged 60 years and above [18]. The two sub-counties of Nansana and Busukuma were purposively selected for their representation of both urban and rural populations.

### Sampling

In this study, sampling was done in several stages. Initially, we purposively selected two sub-counties for the study owing to their large populations and area size. Participants for this study had to be 60 years and above and residents in these two sub-counties. Next, we randomly selected one parish from each sub-county and purposively selected all the villages for inclusion in the study. With the help of the local village leaders known as the Local Councilor One (LC1) and the existing system of Ministry of Health approved village health workers, we were able to understand the layout of the villages and the households therein. Although we were not able to get a list of all villages as a sampling frame, we learnt that the villages were within walking distance from one another. Therefore, by walking down one village road, one was likely to traverse three villages in the process. Thus, we decided to start our village road treks from a central point at the sub-county headquarters. Spanning out in different directions, the research assistants were able to identify and interview study respondents who were 60 years and older from their homesteads lying along the chosen village roads. Research assistants were; a psychiatric nurse, a social worker and a community psychologist who were trained prior to conducting the study trained. The Principal Investigator (PI) also participated in interviewing respondents. The research team exhausted all households along a particular road before returning to the center to pick a new road to follow. The village health team members moved alongside each researcher and acted as the community gatekeepers that introduced the research team, as well as guiding the team on where to stop. We harnessed the intimate knowledge of the villages that the VHTs had such as, knowing specific households where an elderly person resided. We excluded all elderly people who had not lived in that village for more than 12 months and those who could not answer our questions because they were too ill. In each identified household, we interviewed one elderly person who met the study inclusion criteria. We made one call back visit to ensure that we got all potential respondents who may have not been home at the time of our visit.

### Sample size calculation

A sample size of 380 respondents from the two sub-counties was calculated using the Cochran method and the following parameters: an elected alpha of 1.96; an estimated portion of older people in Uganda of 4.6% [19]; q of (1− p); and an estimated margin of error d at

$$n_{\text{O}}={\frac{Z^{2}pq}{e^{2}}}^{0.05.}$$

### Data collection tools

The data collection was conducted by three research assistants who were well-trained on the protocol and research subject protection, and the PI, using structured questionnaires. The questionnaires were first translated into the local language of Luganda by a process of forward, backward and forward translation by two teams of mental health professionals working independently of each other. Any disparities were resolved through consensus.

The study questionnaire had three parts, namely:

- Social Demographic factors - sex, age, marital status, education level and religion;
- A culturally adapted version of the 35 item Older Persons’ QoL Questionnaire (OPQOL) [20]. The OPQOL has been previously validated for studies in Iran [21], and the Czech Republic [22]. The OPQOL is a 5-point Likert scale (strongly disagree to strongly disagree), representing questions about life overall, health, social relationships and participation, independence, control over life, freedom, area (home and neighborhood), psychological and emotional well-being, financial circumstances, religion/culture. For this study, 4 questions of the original version were dropped during the adaptation phase. The tool was then categorized into 3 subcategories of QoL, namely: Health (HE), Social Economic QoL (SE) and the Psychosocial QoL (PS). The HE QoL had 10 questions, SE QoL was represented by 9 questions and the PS QoL represented by 14 questions. 2 questions were dropped because they were covered in the social demographic questions
- A culturally adapted version of the 26- item Brief Ageing Perceptions Questionnaire (B-APQ) [23]. The B-APQ is a 5 point Likert scale form that assesses emotional regulation, perceptions about control of social life and perceptions about physical age. The B-APQ questionnaire has been previously validated for studies in Malaysia [24]. It was adapted for the local Ugandan context for this study

### Statistical analysis

Data were double-entered into Epidata 4.2, cleaned and exported into STATA 15.1/MP for analysis. Data was analyzed using STATA software (StataCorp LP, 4905 Lakeway Drive, College Station, TX, USA). Summary statistics for continuous variables were presented as mean ± standard deviation while categorical variables were presented as frequencies and proportions with their 95% confidence intervals in tables. A Pearson’s Chi-square test was then used to determine the relationship between the perceptions and quality of life. As this was one of the first studies conducted related to QoL in Uganda, a p-value of <0.05 was considered to be significant.

### Ethical consideration

The study was approved by the Makerere School of Health Sciences Review Board (#SHSREC REF: 2017–094) and the Uganda National Council for Science and Technology (HS195ES). Study approval from the participating district was also secured. Informed consent was obtained from the respondents prior to subjecting them to any study tools. Written consent was obtained from respondents who could read and write, while those who could not read and write, we used audio recorded verbal consent. In addition to verbal consent, they were also required to append their thumb prints on the consent paper. Study respondents found to be in significant physical distress were excused and referred to the health center for medical assistance, escorted by one of the Village Health Team members. Respondents were assured of confidentiality before the start of each interview. All data collected was kept under key and lock, and only the research team had access to them. Personal identifiers were not used at data entry.

## Results

Table 1 shows the demographic characteristics of the community population of older persons in Uganda’s communities of Wakiso district. Table 2 displays the Quality-of-life dimension results as shown by each participant on the OPQOL-35 questionnaire. Tables 3& 4 show the distribution of the 3 QoL domains in relation to the demographic characteristics. Table 5 presents s the ageing perceptions results as shown on the B-APQ. Tables 6–8 shows the association of perception score in each domain and specific QOL domain.

### Participant characteristics

A total of 460 eligible older persons were invited to participate, of whom 380 consented to enroll. The mean age was 69.6± 9.1 years, 256 (68%) were female and majority (n = 243, 64%) had primary education. Majority of the participants (n=353, 93%) reported having children and 182 (48%) were currently staying with at least one of their children (Table 1).

### Older persons Quality of life

Generally, the older persons had poor quality of life in all domains. Social economic being the worst with 90% (95% CI: 86–93) of the participants having poor socioeconomic quality of life score, followed by 86% (95% CI: 82–89) of the participants having poor health quality of life score and 83% (95% CI: 78–86) had a poor psychosocial quality of life score (Table 2).

### Distribution of QoL domains in relation to the social demographic characteristics

While relating to each domain; sex (p=0.024), education (p=0.001), employment status (p=0.023) and monthly expenditure (p=0.003) were all significantly associated with the Health related QoL of the participants. Education (p=0.005), employment status (p=0.01) and monthly expenditure (p=0.023) were all significantly associated with the socioeconomic QoL of the participants (Table 3). Only two factors were significantly associated with psychosocial quality of life, marital status (p=0.002) and monthly expenditure in Uganda shillings (p=0.031) (Table 4).

### The brief aging perceptions of the older persons

Majority (n=232, 61%) of the participants had negative overall perceptions towards aging, 329 (86%) had negative perceptions towards physical age, 245 (64%) had negative perceptions towards participation in social activities while 246 (65%) had positive perceptions towards regulation of emotions (Table 5).

### Association of perception score in each domain and specific QOL domain

Using a Pearson’s Chi-square to determine any significant associations between perceptions and QoL for each domain, participation in social activities (p=0.007) and Regulation of emotions (p<0.001) were significantly associated in health QoL domain (Table 6). The same perceptions were significant for social economic QoL domain, participation in social activities (p=0.021) and Regulation of emotions (p=0.001) (Table 7). While Regulation of emotions (p=0.002) was only significant in psychosocial QoL (Table 8).

## Discussion

This was a community-placed survey that employed a cross-sectional study design where a representative sample was drawn from two sub-counties of Wakiso district. Participants were met in their home steads; they were willing to participate and gave full attention to the study. However, this did not come without some challenges. For example, the researcher-administered questionnaire, did not take the expected average, of 30 minutes for most participants. Most times, the participants needed frequent bathroom breaks, and at times some interview questions resonated with them, so they would take some time to share personal stories, like death of loved ones, reasons why they were divorced or separated and often times they shared experiences with health workers. On several occasions, the participants had expectations other than the research objective, such as requesting the research team to lobby for government considerations for older persons’ social economic wellbeing. Participants also shared other personal experiences with the health system and the health workers, constantly alluding to the need for health worker sensitization to learn better communication styles with older patients. This reflects a need to move our research from a community placed to community-based research to get in-depth understanding of the challenges face by older people.

For this study, a total of 380 older persons participated with a mean age of 69.6±9.1 years. This distribution is similar to the Uganda population census distribution of 2018 that showed that majority of older persons’ aged 60 and older are in the age category of 60–69. Further, the study had more females than males which is also accounted for by the Uganda population census distribution of 2018 that showed a higher percentage of women in the 60 and older age bracket. More than half (70%) of our respondents were living below the poverty line with a monthly expenditure of not more than Ugx 200,000 ($110). The Ugandan situation is seen across the low- and middle-income countries that have majority of their population, including older people, living below the poverty line (Ref).

Most of the respondents were either offering social support to grandchildren and children that were still staying at home with their older parents or receiving some form of social and financial support from their children or grandchildren in the form of paying for medical bills and providing food. Traditionally, African societies have been characterized by cultural systems, which gave higher status to older people. In the past, the experience and knowledge that older people offered was recognized and appreciated, which contributed to a sense of integration in the community. This is still the practice in Uganda and is evidenced by having older people taking care of both orphaned and non-orphaned grandchildren. Individual participants noted that this kept them active, though they often noted feeling fatigued by this responsibility. We also see this playing out in the silent expectation that many parents had about receiving some form of support from their children. This became complicated, however, if the children were either dead or not financially able to support their parents. Our study’s findings show that taking care of grandchildren and receiving support from adult children, is still a very much alive practice in these rural areas of Uganda.

In this study, we set out to determine the relationship between perceptions on aging and quality of life among the elderly in Central Uganda. Our respondents reported poor quality of life across all the domains that we measured, including health quality of life. Health related quality of life is a multi- dimensional concept that includes domains related to physical, mental, emotional and social functioning. For this study, all the mentioned domains were considered. Our study found a positive association between health quality of life and older persons’ perception of their ability to participate in social activities, like family gatherings or community events. Our findings complement the findings by Detta, Detta and Majumdar [25], who in their study of role of social interaction on quality of life, posited that available social interactions has a significant role in improving the quality of life of elderly people. Our results also compliment the work by Jalali-Farahani et al. [26], who concluded that perceived social support was found to be both directly and indirectly associated with physical and mental aspects of HRQoL.

The major limitation of this study was its being a community placed study as opposed to a community based. This prevented us from getting in-depth information. We however we were still able to obtain some important information that was outside our original research scope. There is need for community-based studies to obtain a deeper understanding of the pertinent issues affecting Uganda’s older persons’ community.

## Conclusion and Recommendations

In conclusion, there is a positive association between QoL and perceptions of ageing. We recommend the need for more community based and community engaged studies to get a deeper understanding of the older persons’ strengths and barriers in the improvement of QoL across all domains.

## Figures and Tables

**Table 1:** Table of Demographic characteristics at baseline.

Characteristic	Frequency (n %)
Female	259(68)
Male	121 (32)
**Marital status**	
Widowed	164 (43)
Married	119 (31)
Divorced	89 (23)
Single	8 (2)
**Education**	
None	64 (17)
Primary	243 (64)
Secondary	57 (15)
Tertiary	16 (4)
**Employment status**	
Formal	13 (3)
Casual labor	139 (37)
Business	88 (23)
Not employed	134 (35)
**Monthly expenditure (UGX)**	
≤50,000	163 (42)
60,000–100,000	68 (17)
110,000–200,000	32 (8)
>200,000	42 (11)

**Table 2:** Older person’s quality of life as shown on the OPQOL- 35 questionnaire.

QoL	Frequency (n %)	95% Confidence interval	
		Lower limit (%)	Upper limit (%)
**Health related**			
Good	53 (14)		
Poor	327 (86)	82	89
**Social economic**			
Good	37 (10)		
Poor	343 (90)	86	93
**Psychosocial**			
Good	66 (17)		
Poor	314 (83)	78	86

Good QoL = Any score between 35 and 105 on the OPQOL questionnaire

Poor QoL = Any score above 105 on the OPQOL questionnaire

**Table 3:** Health related QoL, socioeconomic QoL and their distribution across the social demographic characteristics.

Characteristic	Health related QoL			Socioeconomic QoL		
	Good	Poor	P-value	Good	Poor	P-value
**Sex**			**0.024** *			**0.410**
Female	29 (54.7)	230 (70.3)		23 (62.2)	236 (68.8)	
Male	24 (45.3)	97 (29.7)		14 (37.8)	107 (31.2)	
**Marital status**			0.060			0.056
Widowed	17 (32.1)	147 (44.9)		13 (35.1)	151 (44.0)	
Married	25 (47.2)	94 (28.8)		19 (51.4)	100 (29.2)	
Divorced	11 (20.8)	78 (23.8)		5 (13.5)	84 (24.5)	
Single	0	8 (2.5)		0	8 (2.3)	
**Education**			**0.001** *			**0.005** *
None	5 (9.4)	59 (18.0)		8 (21.6)	56 (16.3)	
Primary	27 (50.9)	216 (66.1)		16 (43.2)	227 (66.2)	
Secondary	16 (30.2)	41 (12.5)		8 (21.6)	46 (14.3)	
Tertiary	5 (9.4)	11 (3.4)		5 (13.5)	11 (3.2)	
**Employment status**			**0.023** *			**0.010** *
Formal	1 (1.9)	12 (3.7)		4 (11.1)	9 (2.7)	
Casual labor	26 (49.1)	113 (35.2)		13 (36.1)	126 (37.3)	
Business	16 (30.2)	72 (22.4)		12 (33.3)	76 (22.5)	
Not employed	10 (18.9)	124 (38.6)		7 (19.4)	127 (37.6)	
**Monthly expenditure (UGX)**			**0.003** *			**0.023** *
≤50,000	14 (29.8)	149 (57.8)		14 (41.2)	149 (55.0)	
60,000–100,000	16 (34.0)	52 (20.2)		6 (17.6)	62 (22.9)	
110,000–200,000	9(19.1)	23 (8.9)		3 (8.8)	29 (10.7)	
>200,000	8 (17.0)	34 (13.2)		11 (32.4)	31 (11.4)	

* P- Value is significant at <0.05

**Table 4:** Psychosocial QoL and its distribution across the social demographic characteristics.

Characteristic	Psychosocial QoL		
	Good	Poor	P-value
**Sex**			**0.996**
Female	45 (68.2)	214 (68.2)	
Male	21 (31.8)	100 (31.8)	
**Marital status**			**0.002** *
Widowed	27 (40.9)	137 (43.6)	
Married	32 (48.5)	87 (27.7)	
Divorced	7 (10.6)	82 (26.1)	
Single	0	8 (2.6)	
**Education**			**0.308**
None	9 (13.6)	55 (17.5)	
Primary	39 (59.1)	204 (65.0)	
Secondary	14 (21.2)	43 (13.7)	
Tertiary	4 (6.1)	12 (3.8)	
**Employment status**			**0.644**
Formal	2 (3.1)	11 (3.6)	
Casual labor	20 (30.8)	119 (38.5)	
Business	18 (27.7)	70 (22.7)	
Not employed	25 (38.5)	109 (35.3)	
**Monthly expenditure (UGX)**			**0.031** *
≤50,000	19 (37.2)	144 (56.7)	
60,000–100,000	12 (23.5)	56 (22.1)	
110,000–200,000	8 (15.7)	24 (9.5)	
>200,000	12 (23.5)	30 (11.8)	

* P- Value is significant at < 0.05

**Table 5:** The brief aging perceptions as shown on the B-APQ.

Characteristic	Frequency, n (%)
**Overall perceptions towards aging**	
Positive	148 (39)
Negative	232 (61)
**Physical age (negative perception)**	
Agree	51 (14)
Disagree	329 (86)
**Physical age (positive perception)**	
Agree	312 (82)
Disagree	68 (18)
**Participation in social activities (Positive perception)**	
Agree	178 (47)
Disagree	202 (53)
**Participation in social activities (Negative perception)**	
Agree	135(36)
Disagree	245 (64)
**Regulation of emotions**	
Positive perception	246 (65)
Negative perception	134 (35)

Positive perception = Any score 54 and more on the B-APQ

Negative perception = Any score less than 54 on the B-APQ

**Table 6:** Health quality of life and ageing perceptions.

Characteristic	QOL			
	Poor	Good	X^2^	P-value
**Overall perceptions towards aging**				
Negative	111 (98.2)	2 (1.8)	0.0878	0.767
Positive	261 (97.7)	6 (2.3)		
**Physical age (positive perception)**				
Agree	91 (80.5)	22 (19.5)	0.2713	0.602
Disagree	221 (82.8)	46 (17.2)		
**Participation in social activities (Positive perception)**				
Agree	65 (57.5)	48 (42.5)	7.3670	0.007*
Disagree	113 (42.3)	154 (57.7)		
**Regulation of emotions**				
Positive	58 (51.3)	55 (48.7)	18.1803	<0.001*
Negative	76 (28.5)	191 (71.5)		

* statistically significant p-value

**Table 7:** Social economic quality of life and ageing perceptions.

Characteristic	QOL			
	Poor	Good	X^2^	P-value
**Overall perceptions towards aging**				
Negative	37 (100)	0		0.348
Positive	335 (97.7)	8 (2.3)	0.8815	
**Physical age (positive perception)**				
Agree	32 (86.5)	5 (13.5)	0.5355	0.464
Disagree	280 (81.6)	63 (18.4)		
**Participation in social activities (positive perception)**				
Agree	24 (64.9)	13 (35.1)	5.3472	0.021*
Disagree	154 (44.9)	189 (55.1)		
**Regulation of emotions**				
Positive	22 (59.5)	15 (40.5)	10.5128	0.001*
Negative	112 (32.7)	231 (67.3)		

* statistically significant p-value

**Table 8:** Psychosocial quality of life and ageing perceptions.

Characteristic	QOL			
	Poor	Good	X^2^	P-value
**Overall perceptions towards aging**				
Negative	66 (100)	0	1.7177	0.190
Positive	306 (97.4)	8 (2.6)		
**Physical age (positive perception)**				
Agree	56 (84.8)	10 (15.2)	0.4091	0.522
Disagree	256 (81.5)	58 (18.5)		
**Participation in social activities (positive perception)**				
Agree	36 (54.6)	30 (45.4)	1.9035	0.168
Disagree	142 (45.2)	172 (54.8)		
**Regulation of emotions**				
Positive	34 (51.5)	32 (48.5)	9.2414	0.002*
Negative	100 (31.8)	214 (68.2)		

* statistically significant p-value

## Data Availability

The datasets used and analyzed during this study are available from the corresponding author on reasonable request.

## Abbreviations

QoL: Quality of Life
OPQoL: Older Person’s QoL Questionnaire
BAPQ: Brief Ageing Perceptions Questionnaire
UBOS: Uganda Bureau of Statistics
